# Supplementary material for: Cohesin-mediated loop anchors confine the locations of human replication origins
Source: Nature. 2022 Jun 8;606(7915):812–9. doi: 10.1038/s41586-022-04803-0 (PMC9217744; doi:10.1038/s41586-022-04803-0)
Supplement: Supplementary file 1 — This file contains full legends for Supplementary Tables 1–19, Methods, Fig. 1 and References. [file 41586_2022_4803_MOESM1_ESM.pdf]

nature portfolio

https://doi.org/10.1038/s41586-022-04803-0

Supplementary information

Cohesin-mediated loop anchors confine the locations of human replication origins

In the format provided by the authors and unedited

Cohesin-mediated loop anchors confine the location of human replication origins

Supplementary Information

Daniel J. Emerson[1,2,3,#], Peiyao A. Zhao[4, #], Ashley L. Cook[1,2,3, #], R. Jordan Barnett[1,2,3], Kyle Klein[4], Dalila Saulebekova[5], Chunmin Ge[1,2,3], Linda Zhou[1,2,3], Zoltan Simandi[1,2,3], Miriam K. Minsk[1,2,3], Katelyn R. Titus[1,2,3], Weitao Wang[5], Wanfeng Gong[1,2,3], Di Zhang[6], Liyan Yang[7], Sergey V. Venvev[7], Johan H. Gibcus[7], Hongbo Yang[8], Takayo Sasaki[9], Masato T. Kanemaki[10,11], Feng Yue[8], Job Dekker[7, 12], Chun-Long Chen[5], David M. Gilbert[4, 9], Jennifer E. Phillips-Cremins[1,2,3,13,*]

1 Department of Bioengineering, University of Pennsylvania, Philadelphia, PA, USA
2 Epigenetics Institute, Perelman School of Medicine, University of Pennsylvania, USA
3 Department of Genetics, Perelman School of Medicine, University of Pennsylvania, USA
4 Department of Biological Science, Florida State University, USA
5 Institut Curie, PSL Research University, CNRS UMR3244, Dynamics of Genetic Information, Sorbonne Université, 75005 Paris, France
6 Children's Hospital of Pennsylvania, Philadelphia, PA, USA
7 University of Massachusetts Medical School, Worcester, MA, USA
8 Department of Biochemistry and Molecular Genetics, Feinberg School of Medicine, Northwestern University, Chicago, Illinois, 60611, USA
9 San Diego Biomedical Research Institute, San Diego, CA 92121, USA
10 Department of Chromosome Science, National Institute of Genetics, Research Organization of Information and Systems (ROIS), Yata 1111, Mishima, Shizuoka 411-8540, Japan.
11 Department of Genetics, The Graduate University for Advanced Studies (Sokendai), Yata 1111, Mishima, Shizuoka 411-8540, Japan
12 Howard Hughes Medical Institute, USA
13 New York Stem Cell Foundation – Robertson Investigator, USA

# Authors contributed equally to this work
*Corresponding author: jcremins@seas.upenn.edu

Supplementary Tables

Supplementary Table 1. TADs/subTADs genome-wide from Hi-C 2.5 in H1 human ES cells

Supplementary Table 2. Loop calls at three different parameter combinations from Hi-C 2.5 in H1 human ES cells

Supplementary Table 3. Dot and Dotless TADs/subTADs genome-wide from Hi-C 2.5 in H1 human ES cells

Supplementary Table 4. Dot and Dotless TAD/subTAD boundaries genome-wide from Hi-C 2.5 in H1 human ES cells

Supplementary Table 5. Initiation Zones from 16-fraction Repli-seq in H1 human ES cells (Group 1, read depth normalized and scaled, full read depth)

Supplementary Table 6. CTCF and Rad21 ChIP-seq peaks in H1 human ES cells

Supplementary Table 7. Six classes of TAD/subTAD boundaries stratified by CTCF+cohesin co-occupancy and CTCF motif orientation genome-wide in H1 human ES cells

Supplementary Table 8. Twelve classes of TAD/subTAD boundaries stratified by CTCF+cohesin co-occupancy, CTCF motif orientation, and colocalization with transcribed genes in H1 human ES cells

Supplementary Table 9. TADs/subTADs genome-wide from Rao et al. 2017 Hi-C in the untreated HCT116 Rad21-mAID cell line

Supplementary Table 10. Loop calls genome-wide from Rao et al. 2017 Hi-C in the untreated HCT116 Rad21-mAID cell line

Supplementary Table 11. Dot and Dotless TADs/subTADs genome-wide from Rao et al. 2017 Hi-C in the untreated HCT116 Rad21-mAID cell line

Supplementary Table 12. Dot and Dotless TAD/subTAD boundaries genome-wide from Rao et al. 2017 Hi-C in the untreated HCT116 Rad21-mAID cell line

Supplementary Table 13. CTCF and Rad21 ChIP-seq peaks in the wild type HCT116 parent cell line

Supplementary Table 14. Six classes of TAD/subTAD boundaries stratified by CTCF+cohesin co-occupancy and CTCF motif orientation genome-wide from Rao et al. 2017 Hi-C in the untreated HCT116 Rad21-mAID cell line

Supplementary Table 15. Initiation zones called in wild type human HCT116 cells (Group 2, mitochondria-normalized, full read depth)

Supplementary Table 16. Initiation zones called in wild type human HCT116 cells and HCT116 Rad21-mAID auxin-treated knock-down cells (Group 3, read depth normalized with threshold, down sampled to lowest fraction read depth)

Supplementary Table 17. Initiation Zones called in wild type human HCT116 cells and HCT116 WAPL-mAID2 auxin-treated knock-down cells (Group 4, read depth normalized with threshold, down sampled to lowest fraction read depth)

Supplementary Table 18. Loop calls genome-wide in untreated and auxin-treated HCT116 WAPL-mAID2 cells

Supplementary Table 19. 3D genome excluded regions in H1 human ES cells

Supplementary Methods

Domain calling

We analyzed Hi-C data for Tier 1 H1 Human hES (hES) cells created in Phase 1 of the 4D Nucleome[52]. We identified TADs/subTADs genome-wide as previously described using 3DnetMod (https://bitbucket.org/creminslab/cremins_lab_tadsubtad_calling_pipeline_11_6_2021/src/master/)[53-57] with parameters optimization on balanced and merged 10 kb binned Hi-C matrices for H1 human ES cells (hg38) from the 4DNucleome consortium (https://data.4dnucleome.org/files-processed/4DNFI82R42AD/), as well as 10 kb binned, Knight-Ruiz balanced Hi-C matrices for untreated HCT116 Rad21-mAID cells (HCT116 WT; hg38) (https://data.4dnucleome.org/files-processed/4DNFIFLDVASC/)[58].

We log transformed genome-wide counts data for each merged replicate and chunked into 6 Mb regions with 4 Mb overlap as well as 3 Mb regions with 2 Mb overlap. We analyzed all chunked regions with sufficient reads, and excluded sparse regions that exhibited either (i) consecutive zero counts on the diagonal for a genomic distance of ≥ 500 kb or (ii) zero counts for ≥ 1/3 of all pixels on diagonal of the chunked region. We identified plateaus of consecutive gammas, in 0.01 gamma steps, that show the same number of domains (mean per 20 partitions) and selected a minimum consecutive gamma plateau size of 16 for 6 Mb chunked regions and for 3 Mb chunked regions either plateau size of 6 for hES Hi-C, or 8 for HCT116. To run 3DNetMod optimally, a sweep of gamma values was computed as the mean gamma at every plateau. We identified the optimal genomic location of domains at each gamma by running 3DNetMod 20 times (i.e. 20 partitions) and computing the consensus genomic location of domains from the 20 partitions via the adjusted rand index. We then filtered domains smaller than 60 kb for hES, or 80 kb for HCT116, from the genome-wide list. Moreover, we removed domains within 20 bins from the edges of chunked regions and merged all regions to create a concatenated list of all genome-wide domains after filtering steps from both the 6 Mb and 3 Mb chunked regions. To account for redundant, nearly fully overlapping domains, those that colocalized +/- 70 kb with both boundaries were merged into a single domain. The merged domain was bounded by start and end coordinates separated by the largest genomic distance. Finally, we established a set of domains with unique boundary locations by adjusting boundaries to ensure that domains with similar genomic coordinates shared a single consistent boundary. We iterated through all domains assessing left and right boundaries separately. If two or more domains come into close contact (i.e. the gap between them is smaller than 7.5% of the domain size for all adjacent domains or within 70 kb), then the adjacent close boundaries are adjusted to an average boundary.

Compartment calling

To identify A/B compartments[57, 59, 60], we performed eigenvector decomposition per chromosome on each balanced cis Hi-C matrix at 25 kb matrix resolution for hES and 25 kb matrix resolution for untreated and auxin-treated HCT116 WAPL-mAID2 cells. We normalized the balanced matrix by a global expected distance dependence mean counts value, followed by removal of rows and columns with less than 2% non-zero counts coverage. We transformed each off-diagonal count to a z-score and computed a Pearson correlation matrix from the z-score. We then performed eigenvector decomposition on the z-scored Pearson correlation matrix using LA.eig() (linalg package in numpy), and selected the eigenvector with the largest eigenvalue. Inflection points demarcating boundaries of compartments were computed by identifying genomic coordinates with a transition in eigenvector sign. Compartments on each chromosome were subsequently assigned either an A or B identity by collecting compartment intervals of same eigenvector sign orientation (positive or negative) and counting total number of unique genes for each direction then reassigning those with greater gene number intersection as A and the lesser as B.

Loop calling: Expected modeling

We built a dot calling algorithm for Hi-C data genome-wide based on previously published work by our group and Aiden and colleagues with several modifications (https://bitbucket.org/creminslab/cremins_lab_loop_calling_pipeline_11_6_2021/src/initial/)[55, 61-68]. We restricted all dot calling to bin-bin interaction pair distances ≤ 10 Mb. For published hES Hi-C 2.5[52] and wild type HCT116 (HCT116 Rad21 WT; untreated HCT116 Rad21-mAID) Hi-C[58], we computed the expected background on merged, balanced counts matrices binned at 10 kb. For wild type HCT116 (HCT116 WAPL WT; untreated HCT116 WAPL-mAID2) and HCT116 WAPL knock-down (HCT116 WAPL KD; auxin-treated HCT116 WAPL-mAID2), we computed the expected background on merged, balanced counts matrices binned at 20 kb.

First, a one-dimensional distance-dependent expected model, D, was computed by averaging interaction count of each of the first 1,000 (hES and HCT116 Rad21 WT) or 500 (HCT116 WAPL WT and HCT116 WAPL KD) diagonals spanning 10 Mb (**Equation 1** shown for hES and HCT116 Rad21 WT):

$$D_d = \underset{b-a=d}{\text{gmean}}\left(S_{a,b}\right) \ \forall\ d \text{ such that } 0 \leq d \leq 1{,}000 \tag{1}$$

where D_d is the expected value for the interaction between two bins separated by d bins, S is the balanced contact matrix, and the geometric mean is computed over sets of bin-bin pairs separated by the same number of bins, $b - a = d$, with pseudocount 1 added to each count. We then incorporated the distance correction factor D_d into an overall donut-corrected expected value as in Rao et al. and our own previous work[55, 61-68] (**Equation 2**):

$$E_{i,j}^{DF} = D_{i,j} \times \frac{\sum_{(a,b)\in DF_{i,j}} S_{a,b}}{\sum_{(a,b)\in DF_{i,j}} D_d} \tag{2}$$

where $E_{i,j}^{DF}$ represents the matrix of expected values corrected using the donut footprint. The donut filter summation factor corrects the one-dimensional distance-dependent expected value $D_{i,j}$ at pixel i, j to account for local enrichment or depletion of balanced values $S_{a,b}$ in the local donut-shaped window relative to their own one-dimensional distance-dependent expected values D_d. Points below the diagonal or beyond maximum interaction distance of 10 Mb were excluded from contributing to any of the summations.

For all Hi-C data in HCT116 cells (HCT116 Rad21 WT, HCT116 WAPL WT, and HCT116 WAPL KD), we computed a local expected background interaction frequency by applying geometrical donut, lower left, and vertical/horizontal filters[61] with defined p/w parameters at distance scales of either > 200 kb (HCT116 Rad21 WT) or at distance scale > 400 kb (HCT116 WAPL WT and HCT116 WAPL KD) and a geometrical upper-triangle filter (so-called triu filter) with defined p/w parameters at distance scales ≤ 200 kb (400 kb HCT116 WAPL WT and HCT116 WAPL KD). For Hi-C 2.5 data in hES cells, we applied the geometrical donut, lower left, and vertical/horizontal filters with a defined set of p/w parameters at all distance scales. Mathematically, the local donut footprint is represented according to (**Equation 3**):

$$\begin{aligned} DF_{i,j} = \{(a,b) \mid (|a-i| \leq w) \wedge (|b-j| \leq w) \wedge\ (a \neq i) \wedge (b \neq j) \\ \wedge \left((|a-i| > p) \vee (|b-j| > p)\right)\} \end{aligned} \tag{3}$$

where $DF_{i,j}$ represents the set of bin-bin pairs (a, b) that are included in the donut footprint centered on bin-bin pair (i, j), and p and w are parameters that control the inner and outer radius of the donut shape. Bin-bin pairs (a, b) were included in the donut footprint around bin-bin pair (i, j) if they lay within a $(2w + 1) \times (2w + 1)$ square centered on (i, j) unless (i) they fell on the same row or same column as (i, j) or (ii) they lay within a $(2p + 1) \times (2p + 1)$ square centered on (i, j). For published hES and HCT116 Rad21 WT, we used $p = 2 \,/\, w = 10$ and $p = 4 \,/\, w = 10$, respectively. For HCT116 WAPL WT and HCT116 WAPL KD, we used $p = 4 \,/\, w = 16$.

We employed a lower left footprint, as proposed by Rao et al.[57], which contained only points of the donut footprint that lay both below and to the left of each (i, j)th pixel (**Equation 4**):

$$LLF_{i,j} = \{ (a, b) \in DF_{i,j} \mid (a < i) \wedge (b < j) \} \quad \textbf{(4)}$$

where $LLF_{i,j}$ represents the set of bin-bin pairs (a, b) that are included in the lower left footprint centered on bin-bin pair (i, j). To compute the lower left footprint $LLF_{i,j}$, only those points that lay below and to the left of the (i, j)th pixel in the original donut filter were kept. The lower left footprint was used to compute lower left corrected expected values (**Equation 5**):

$$E_{i,j}^{LLF} = D_{i,j} \times \frac{\sum_{(a,b) \in LLF_{i,j}} S_{a,b}}{\sum_{(a,b) \in LLF_{i,j}} D_d} \quad \textbf{(5)}$$

We implemented vertical and horizontal footprints to account for the presence of stripes, indicative of loop extrusion. The presence of a loop extrusion results in a stripe of enrichment within a TAD. Without vertical and horizontal filters, many false positives are called along the length of the stripe. The footprint for the horizontal filter centered around bin-bin pair (i, j) is described mathematically by (**Equation 6**):

$$HF_{i,j} = \{(a, b) \mid \big((a = i - 1) \vee (a = i) \vee (a = i + 1)\big) \wedge (|b - j| > p) \wedge (|b - j| \leq w)\} \quad \textbf{(6)}$$

where $HF_{i,j}$ represents the set of bin-bin pairs (a, b) that are included in the horizontal footprint centered on bin-bin pair (i, j). The corrected expected values as predicted by the horizontal filter were computed by (**Equation 7**):

$$E_{i,j}^{HF} = D_{i,j} \times \frac{\sum_{(a,b) \in HF_{i,j}} S_{a,b}}{\sum_{(a,b) \in HF_{i,j}} D_d} \quad \textbf{(7)}$$

The vertical footprint is similarly determined using (**Equation 8**):

$$VF_{i,j} = \{(a, b) \mid \big((b = j - 1) \vee (b = j) \vee (b = j + 1)\big) \wedge (|a - i| > p) \wedge (|a - i| \leq w)\} \quad \textbf{(8)}$$

where $VF_{i,j}$represents the set of bin-bin pairs (a, b) that are included in the vertical footprint and centered around the bin-bin pair (i, j). The vertical footprint was then used to compute the vertical corrected expected values. (**Equation 9**):

$$E_{i,j}^{VF} = D_{I,j} \times \frac{\sum_{(a,b)\in VF_{i,j}} S_{a,b}}{\sum_{(a,b)\in VF_{i,j}} D_d} \quad \textbf{(9)}$$

The maximum of **Equations 2, 5, 7**, and **9** was used for the final corrected expected value for all bin-bin pairs in hES, and for all bin-bin pairs > 200 kb in HCT116 RAD21 WT or > 400 kb for HCT116 WAPL WT and HCT116 WAPL KD.

To ensure sensitive detection of corner-dots close to the high-count diagonal, we implemented an upper triangular donut footprint (termed triu) alone to model expected values in all HCT116 cell lines for all bin-bin pairs in the distance range of ≤ 200 kb for HCT116 Rad21 WT or ≤ 400 kb for HCT116 WAPL WT and HCT116 WAPL KD. We found that close to the diagonal, the lower-left filter was dominating the signal and caused the loss in detection of the majority of directly on-diagonal corner-dots. Specifically, the expected value was overestimated by the donut filter and lower-left filter, thus reducing the sensitivity of dot calling near the diagonal of the contact matrix, in particular for the HCT116 cell type. The upper triangle donut footprint (triu) only includes those bin-bin pairs (a, b) in the donut footprint that have interaction distances greater than or equal to the interaction distance of the entry for which the corrected expected value was computed (**Equation 10**):

$$UTF_{i,j} = \left\{ (a,b) \in DF_{i,j} \mid b - a \geq j - i \right\} \quad \textbf{(10)}$$

where $UTF_{i,j}$ represents the set of bin-bin pairs (a, b) that are included in the upper triangular footprint centered on bin-bin pair (i, j).

We calculated the upper triangular corrected expected value using the triu upper triangular footprint according to (**Equation 11**):

$$E_{i,j}^{UTF} = D_{i,j} \times \frac{\sum_{(a,b)\in UTF_{i,j}} S_{a,b}}{\sum_{(a,b)\in UTF_{i,j}} D_d} \quad \textbf{(11)}$$

For published HCT116 Rad21 WT cells, we used $p = 4$ / $w = 10$ for the triu parameters in the distance range ≤ 200 kb. For HCT116 WAPL WT and HCT116 WAPL KD, we used $p = 4$ / $w = 16$ for the triu parameters in the distance range ≤ 400 kb. The final corrected expected value for bin-bin pairs, ≤ 200 kb in HCT116 Rad21 WT or 400 kb in HCT116 WAPL WT and HCT116 WAPL KD, was determined using **Equation 11**. For values greater than the triu range, the maximum of **Equations 2, 5, 7**, and **9** was used for the final corrected expected value for all HCT116 cell types.

Thus, for all HCT116 cell types, the final expected values $E_{i,j}$ are computed from the upper triangle (triu) expected values for bin-bin pair interaction distances less than or equal to 200 kb, or 400kb, and from the maximum of the other corrected expected values for bin-bin interaction distances greater than either 200 kb, or 400 kb (**Equation 12**):

$$E_{i,j} = \begin{cases} E_{i,j}^{UTF}, & for\ b - a \leq 20 \\ max\left(E_{i,j}^{DF}, E_{i,j}^{LLF}, E_{i,j}^{HF}, E_{i,j}^{VF}\right), & for\ 20 < b - a < 1{,}000 \end{cases} \quad \textbf{(12)}$$

The limit of 1,000 was used for hES and HCT116 Rad21 WT due to 10 kb matrices and 500 for HCT116 WAPL WT and HCT116 WAPL KD due to 20 kb matrices.

The hES final expected values $E_{i,j}$ are computed from the max of the donut filter, lower left filter, and vertical and horizontal filters (**Equation 13**):

$$E_{i,j} = max\left(E_{i,j}^{DF}, E_{i,j}^{LLF}, E_{i,j}^{HF}, E_{i,j}^{VF}\right) \quad \textbf{(13)}$$

Loop calling: P-values

The final expected value $E_{i,j}$ and the balanced bias vector c was used to compute a biased expected value for comparison to the raw read counts $X_{i,j}$ (**Equation 14**):

$$E_{i,j}^{\text{biased}} = E_{i,j} \times c_i \times c_j \quad \textbf{(14)}$$

A p-value matrix $P_{i,j}$ was then computed against the null hypothesis that the raw read count $X_{i,j}$ was less than or equal to the biased expected value $E_{i,j}^{\text{biased}}$. Specifically, probability that the raw read count $X_{i,j}$ was less than or equal to a Poisson-distributed random variable $X'_{i,j}$ with mean $E_{i,j}^{\text{biased}}$ was computed (**Equation 15**):

$$P_{i,j} = P\left(X_{i,j} \leq X'_{i,j}\right); \quad X'_{i,j} \sim \text{Poisson}\left(E_{i,j}^{\text{biased}}\right) \quad \textbf{(15)}$$

Loop calling: Multiple testing correction

We applied the lambda-chunking strategy from Aiden and colleagues[61] for multiple testing correction involving each bin-bin pair within interaction distance up to 10 Mb. First, bin-bin pairs (i, j) were stratified according to their biased expected values $E_{i,j}^{\text{biased}}$ using logarithmically spaced bins with a bin spacing $2^{1/3}$. This was followed by a Benjamini-Hochberg false discovery rate control for the p-values $P_{i,j}$ for each chunk separately to obtain a matrix of q-values $Q_{i,j}$, which represent the maximum false discovery rate (FDR) at which an interaction would be called significant.

Loop calling: Clustering

After we computed the matrix of q-values $Q_{i,j}$, we identified clusters of nearby significant bin-bin pairs to account for dots composed of multiple nearby pairs. First, an initial set of significant bin-bin pairs were identified using: (1) a q-value $Q_{i,j} \leq 0.025$ (false discovery rate of 2.5%) and a balanced contact value $S_{i,j} \geq 8$ for hES, (2) a q-value $Q_{i,j} \leq 0.3$ and a balanced contact value $S_{i,j} \geq 6$ for HCT116 Rad21 WT (untreated HCT116 Rad21-mAID) cells, and (3) a q-value $Q_{i,j} \leq 0.3$ and a balanced contact value $S_{i,j} \geq 8$ for wild type HCT116 (HCT116 WAPL WT; untreated HCT116 WAPL-mAID2) and HCT116 WAPL knock-down (HCT116 WAPL KD; auxin-treated HCT116 WAPL-mAID2). To further reduce the possibility of false positives, clusters were removed with fewer than three significant bin-bin pairs for all cell types and conditions.

Large "superclusters", composed of smaller dots more likely to represent individual looping interactions, were found in initial calls. Therefore, large clusters were split by applying progressively more stringent q-value thresholds (in order-of-magnitude steps from 0.025 to 1e-20 FDR for hES, 0.3 to 1e-5 for HCT116 Rad21 WT, and 0.3 to 1e-20 for HCT116 WAPL WT and HCT116 WAPL KD). For each more stringent q-value threshold, bin-bin pairs were re-clustered into smaller ones that passed new, more stringent q-value threshold, recursively testing against more stringent q-values until at least a 3-bin cluster

remained. Finally, to further reduce the possibility of false positive interactions being called near the diagonal of the contact matrix, all refined clusters containing a bin-bin pair whose interaction distance was within 3 bins of diagonal were removed.

Loop calling: Single-variable changes in the statistical framework or parameters in Extended Data Figure 4 and their effect on biological conclusions

To assess how our biological conclusions are influenced by loop calling parameters, we called dots genome-wide in hES Hi-C 2.5 data across a range of ten single-variable changes in the statistical framework or parameters (**Extended Data Figure 4**). Coordinates for Options D, F, and H are provided in **Supplementary Tables 2.**

- Option A (N=280,873 loops on all autosomes) shows the effect of only using a global one-dimensional distanced-dependent expected model (see **Equation 1**). This expected model fails to consider local TAD/subTAD structure, resulting in a large number of non-specific, false-positive loops, and is not considered a plausible expected model for dot-calling. An FDR range from $Q_{i,j} \leq 0.025$ to 1e-20 is used.
- Option B (N=41,091 loops on all autosomes) switches the global 1D expected from Option A to a geometrical donut filter with $p = 2$ / $w = 10$ parameters at distance scales > 200 kb and a geometrical upper-triangle filter (so-called TriU filter) with $p = 2$ / $w = 10$ parameters at distance scales ≤ 200 kb (**Equations 2, 5**). While Option B significantly improves the accuracy of loop calls, it fails to account for stripes caused by loop extrusion. An FDR range from $Q_{i,j} \leq 0.025$ to 1e-20 is used.
- In Option C (N=23,040 loops on all autosomes), we added geometrical vertical and horizontal filters to the geometrical donut filter with $p = 2$ / $w = 10$ parameters in Option B to model both local TADs/subTADs and stripes in the expected background > 200 kb (**Equations 7, 9**), as well as a geometrical upper-triangle filter (so-called TriU filter) with $p = 2$ / $w = 10$ parameters at distance scales ≤ 200 kb. An FDR range from $Q_{i,j} \leq 0.025$ to 1e-20 is used.
- In Option D (N=16,248 loops on all autosomes), we remove TriU from Option C, and thus a geometrical donut filter and vertical and horizontal filters with $p = 2$ / $w = 10$ parameters are applied at all distance scales. An FDR range from $Q_{i,j} \leq 0.025$ to 1e-20 is used.
- In Option E (N=18,314 loops on all autosomes), we adjusted the donut filter size in Option D by increasing p to 4 at all distance scales. An FDR range from $Q_{i,j} \leq 0.025$ to 1e-20 is used.
- In Option F (N=22,437 loops on all autosomes) we increased the donut filter size in Option E to $p = 14$. An FDR range from $Q_{i,j} \leq 0.025$ to 1e-20 is used.
- In Option G (N=24,620 loops on all autosomes) we increased the donut filter size in Option E to $p = 16$. An FDR range from $Q_{i,j} \leq 0.025$ to 1e-20 is used.
- In Option H (N=27,415 loops on all autosomes), we re-introduced the TriU filter with $p = 4$ / $w = 16$ at distance scales ≤ 200 kb and with $p = 4$ / $w = 16$ donut expected and vertical/horizontal expected filters at > 200 kb distance scales to Option G. An FDR range from $Q_{i,j} \leq 0.025$ to 1e-20 is used.
- In Options I (N=28,663 loops on all autosomes) and J (N=29,933 loops on all autosomes), we modified Option H by using q-value thresholds of $Q_{i,j} \leq 0.1$, and $Q_{i,j} \leq 0.3$ for Option I and Option J, respectively, to decrease dot calling stringency.

Supplementary Table 2 summarizes the parameters used for each set of hES Hi-C 2.5 loop calls. We then stratified each of these loop calls (Options A-H) into our boundary classes and recomputed the IZ statistical test (**Extended Data Figure 4**). Most importantly, all biological findings from our manuscript remained robust across a full sweep of loop calling stringencies. Option D (N=16,248 loops) – our conservative loop calling set – is indicated by a teal box and was used for the analysis in the main paper. We provide Options D, F, and H as our recommended conservative (D), intermediate (F), and permissive (H) genome-wide loop call sets for use by the scientific community (**Supplementary Table 2, Tab Option D, Tab Option F, and Tab Option H).**

Cut & Run and ChIP-seq processing
We mapped paired-end reads for CTCF Cut & Run in H1 human ES cells (https://data.4dnucleome.org/experiment-set-replicates/4DNES1RQBHPK/) to the human hg38 reference genome using bowtie. We filtered mapped reads to remove optical and PCR duplicates, and merged replicates. We called peaks with Model-based Analysis for ChIP Sequencing v2.0 (MACS2) using a p-value cutoff of $p < 1e\text{-}8$ with punctate peak calling parameters (**Supplementary Table 6**). We downloaded mapped reads for Rad21 and input control ChIP-seq in H9 human ES cells (https://www.ncbi.nlm.nih.gov/geo/query/acc.cgi?acc=GSE105028/). We filtered published mapped reads to remove optical and PCR duplicates and called peaks with MACS2 with a p-value cutoff of $p < 1e\text{-}8$ and broad peak parameters (**Supplementary Table 6**). We downloaded published Rad21 and CTCF ChIP-seq for wild type HCT116 cells from (https://www.encodeproject.org/experiments/ENCSR000BSB/; https://www.encodeproject.org/experiments/ENCSR000BSE/). We aligned reads for both replicates and input control to the hg38 reference genome using bowtie, filtered duplicates and unmapped reads, merged replicates, and called peaks using MACS2 at a p-value cutoff of 1e-6 and broad peak parameters for Rad21 and 1e-8 and narrow peak parameters for CTCF (**Supplementary Table 13**).

RNA-seq processing
We mapped H1 human ES RNA-seq (https://www.encodeproject.org/experiments/ENCSR537BCG/) to the human hg38 reference genome using kallisto (version 0.45.0) then quantified the kallisto results using DEseq2 (R version 4.0.4). We matched the resulting transcripts and their expression (transcripts per million) to their transcript start site (TSS) using biomaRt to produce a list of active and inactive TSS sites. We then used this list to qualify hES TAD/subTAD boundaries as containing active transcription (at least one TSS with TPM > 0; + transcription) or no active transcription (no TSS with TPM > 0; - transcription) within +/- 100 kb of the boundary.

Stratification TAD/subTAD boundary classes
We classified boundaries based on their colocalization with CTCF and Rad21 binding sites, CTCF motif orientation, and the presence of Dot or Dotless TADs/subTADs on one or both sides of the boundary. First, we stratified TADs/subTADs into Dot and Dotless domains. We classified Corner-Dot TADs/subTADs as those in which dots intersect the midpoint (i.e. apex of the TAD triangle) +/- 20% the size of the domain. All others were classified as Dotless TADs/subTADs. Second, we stratified boundaries into Dot and Dotless boundaries. We defined Dot boundaries as those with Dot TADs/subTADs on both (Double-Dot boundaries) or one side (Single-Dot boundaries). We defined Dotless boundaries as those with Dotless TADs/subTADs on both sides.

For all analyses, boundaries were 200 kb. Dotless boundaries overlapping Dot boundaries were removed from consideration. Within Dot boundaries, Single-Dot boundaries overlapping Double-Dot

boundaries were removed from consideration. We used coordinates of 1 bp +/- 100 kb for boundaries demarcating domains adjacent to each other with a shared end coordinate (left domain) and start coordinate (right domain). We annotated from final domain adjusted boundaries with an averaged boundary +/- 100 kb if the gap was less than 7.5% the size of domains on either side or ≤ 70 kb.

We stratified Dot and Dotless boundaries into six classes based on the number of colocalized CTCF and Cohesin binding sites and the orientation of CTCF motifs that intersect these binding sites. We obtained CTCF motifs from JASPAR (https://ccg.epfl.ch//pwmtools/pwmscan.php; JASPAR Core 2020 vertebrate library, motif: CTCF MA0139.1; p-value < 1e-5). We identified colocalized CTCF and cohesin binding sites using bedtools intersect and assigned each CTCF+cohesin site to (+) strand motif orientation, (-) strand motif orientation, or both. We were unable to classify boundaries if they contained CTCF+cohesin binding sites without corresponding CTCF motifs. We stratified boundaries into six mutually exclusive classes used in **Figures 1, 2**, and **3**:

**Boundary classes**	**Structural features**	**Architectural protein profile**	**CTCF motif orientation at colocalized CTCF/cohesin peaks**
Class 1	Dot	2+ CTCF+cohesin colocalized binding sites*	2+ complex orientation
Class 2	Dot	1+ CTCF+cohesin colocalized binding sites	2+ tandem orientation or single motif
Class 3	Dot	0 CTCF+cohesin colocalized binding sites	0 motifs
Class 4	Dotless	2+ CTCF+cohesin colocalized binding sites*	2+ complex orientation
Class 5	Dotless	1+ CTCF+cohesin colocalized binding sites	2+ tandem orientation or single motif
Class 6	Dotless	0 CTCF+cohesin colocalized binding sites	0 motifs

*or edge case of single CTCF+cohesin site with multiple motifs in complex orientation

We further stratified each of our six boundary classes into (a) those with 1 or more transcribed genes and (b) those with no genes or no transcribed genes:

**Boundary classes**	**Structural features**	**Architectural protein profile**	**CTCF motif orientation at colocalized CTCF/cohesin peaks**	**TSS of actively transcribed genes**
Class 1a	Dot	2+ CTCF+cohesin colocalized binding sites*	2+ complex orientation	+
Class 1b	Dot	2+ CTCF+cohesin colocalized binding sites*	2+ complex orientation	-
Class 2a	Dot	1+ CTCF+cohesin colocalized binding sites	2+ tandem orientation or single motif	+

Class 2b	Dot	1+ CTCF+cohesin colocalized binding sites	2+ tandem orientation or single motif	-
Class 3a	Dot	0 CTCF+cohesin colocalized binding sites	0 motifs	+
Class 3b	Dot	0 CTCF+cohesin colocalized binding sites	0 motifs	-
Class 4a	Dotless	2+ CTCF+cohesin colocalized binding sites*	2+ complex orientation	+
Class 4b	Dotless	2+ CTCF+cohesin colocalized binding sites*	2+ complex orientation	-
Class 5a	Dotless	1+ CTCF+cohesin colocalized binding sites	2+ tandem orientation or single motif	+
Class 5b	Dotless	1+ CTCF+cohesin colocalized binding sites	2+ tandem orientation or single motif	-
Class 6a	Dotless	0 CTCF+cohesin colocalized binding sites	0 motifs	+
Class 6b	Dotless	0 CTCF+cohesin colocalized binding sites	0 motifs	-

*or edge case of single CTCF+cohesin site with multiple motifs in complex orientation

Hi-C aggregate heatmap visualization at boundaries

We visualized Hi-C counts around our six boundary classes by stretching to the smallest adjacent domains on each side to a defined length L. Each boundary was used once in the visualization and 60% of the size of the domains was added to the edges of the maps. The counts in every pixel were normalized by mean distance dependence expected value and then averaged across all 2D matrices. We resized with the resize() method in OpenCV image package (https://pypi.org/project/opencv-python/). Hi-C pileups were performed for H1 human ES (hES; https://data.4dnucleome.org/files-processed/4DNFI82R42AD/), wild type HCT116 (HCT116 WT; untreated HCT116 Rad21-mAID cells; https://data.4dnucleome.org/files-processed/4DNFIFLDVASC), and HCT116 Rad21 knock-down (HCT116 Rad21 KD; 6 hour auxin-treated HCT116 Rad21-mAID; https://data.4dnucleome.org/files-processed/4DNFILP99QJS)[58].

Boundary strength with insulation score

We applied a 120 kb square summation window with 20 kb offset to compute an insulation score genome-wide in balanced Hi-C data[69]. Insulation score values at beginning of chromosome corresponding to insufficient counts were discarded as NaN. We constructed mean insulation score pileups at boundaries (+/- 760 kb around the center of each boundary) for each of the six boundary categories and all cell-types.

High-resolution 16-fraction Repli-seq of synchronized RAD21 or WAPL depleted cells

Details of synchronization can be found in Klein et. al.[70]. For HCT116 WAPL-mAID2 cells the endogenous WAPL gene was modified to express WAPL-mAID-Clover in HCT116 cells expressing OsTIR1(F74G) as previously reported[71]. Briefly, either HCT116 RAD21-mAID or HCT116 WAPL-mAID2 cells were synchronized in mitosis via 100 ng/mL nocodazole treatment for 4 hours and mitotic cells were collected by shake off. The percentage of mitotic cells was estimated using metaphase spreads. Collected cells were released into fresh media and 500 μM 3-Indoleacetic acid (Auxin, Sigma, Cat# I2886) was added 30

minutes after release to degrade Rad21. We waited 30 minutes before adding auxin to allow cells to enter G1. 400 μM BrdU was added to the media at 3.5, 5.5, 7.5, and 9.5 hours after release into G1 phase and cells were collected 30 minutes after BrdU addition and fixed in ethanol. FACS analysis was performed as in Oldach and Nieduszynski[72]. Equal numbers of cells from each time point were pooled together for sorting into 16 fractions of S phase DNA content for high-resolution Repli-seq.

Sixteen-fraction Repli-seq visualization

All 16-fraction Repli-seq data were averaged for each fraction at +/- 750 kb intervals around the center of each boundary to give an aggregate heatmap of 16 fractions in each of the six classes. For tandem motif orientation, pileup was reoriented for a leftward (negative) realignment of all motifs.

High-resolution 16-fraction Repli-seq analysis

High-Resolution 16-fraction Repli-seq was analyzed as described in Zhao et al. 2020[73] with modifications as described below:

>Group 1: Sixteen-fraction Repli-seq in H1 human ES cells

For IZ calling in H1 human ES cells in **Figures 1, 4** and **Extended Data Figures 1-6**, we used published Repli-seq raw data (https://data.4dnucleome.org/experiment-sets/4DNESXRBILXJ/) and the Repli-Seq normalized and scaled data array (https://data.4dnucleome.org/files-processed/4DNFI3N8GHKR/). Briefly, raw counts in each fraction ($S_{i,j}$) were normalized by sequencing depth by virtue of read per million (RPM) such that $S_{norm,j,50kb\_bin} = S_j, 50_{kb\_bin} / S_{i,j} * 1e6$. Repli-seq arrays were subsequently constructed from RPM bedgraphs to form 16 rows with each row representing an S phase fraction and each column representing a 50 kb bin. The array was smoothed by applying a Gaussian filter and scaled such that each column sums to 100. This array was used for IZ calling. Autosomal and chromosome X IZs are provided in **Supplementary Table 5** and provided on the 4DN portal (https://data.4dnucleome.org/files-processed/4DNFIRF7WZ3H/).

For visualization in H1 human ES cells in **Figure 1** and **Extended Data Figures 1-3 and 6**, we used published Repli-seq raw data (https://data.4dnucleome.org/experiment-sets/4DNESXRBILXJ/) and the Repli-Seq normalized data array (https://data.4dnucleome.org/files-processed/4DNFIEEYFQ7C/) Briefly, raw counts in each fraction ($S_{i,j}$) were normalized by sequencing depth by virtue of read per million (RPM) such that $S_{norm,j,50kb\_bin} = S_j, 50_{kb\_bin} / S_{i,j} * 1e6$. Repli-seq arrays were subsequently constructed from RPM bedgraphs to form 16 rows with each row representing an S phase fraction and each column representing a 50 kb bin. The array was smoothed by applying a Gaussian filter. This array was used for Repli-seq visualization.

>Group 2: Sixteen-fraction Repli-seq in stand-alone wild type HCT116

For IZ calling in wild type HCT116 used for **Figure 2f** analyses of ORM data, we used published Repli-seq data (https://data.4dnucleome.org/experiment-sets/4DNESNGZM5FG/) and the Repli-Seq mitochondrial normalized data array (https://data.4dnucleome.org/files-processed/4DNFIPIQTMJ9/). We normalized the raw counts in each fraction ($S_{i,j}$) with mitochondrial DNA. For mitochondrial normalization, we computed S_{mj} as the number of reads aligned to mitochondrial DNA where *m* denotes mitochondria and *j* represents S phase fractions 1 through 16. We also computed S_k as the lowest number of aligned mitochondrial reads across all fractions. We computed normalized Repli-seq signal S_{norm} as $S_{norm,j} = S_{i,j}/(S_{mj}/S_k)$. Counts in each fraction ($S_{i,j}$) were then normalized by sequencing depth by virtue of read per million (RPM) such that $S_{norm,j,50kb\_bin} = S_j, 50_{kb\_bin} / S_{i,j} * 1e6$. Repli-seq arrays were subsequently constructed from RPM bedgraphs to form 16 rows with each row representing an S phase fraction and each

column representing a 50 kb bin. The array was smoothed by applying a Gaussian filter and scaled such that each column sums to 100. These full-read-depth, mitochondria-normalized WT HCT116 IZs were used to intersect with the six boundary classes to examine the ORM signal in **Figure 2e**. IZs are provided in **Supplementary Table 15** and provided on the 4DN portal for all autosomal chromosomes (https://data.4dnucleome.org/files-processed/4DNFI95K53YS/).

>Group 3: Sixteen -fraction Repli-seq in wild type HCT116 versus HCT116 Rad21 KD

For IZ calling in **Extended Data Figure 10b-c** analyses, we normalized wild type HCT116 (https://data.4dnucleome.org/experiment-sets/4DNESNGZM5FG/), HCT116 Rad21-mAID knock-down (HCT116 Rad21 KD; auxin-treated HCT116 Rad21-mAID) (https://data.4dnucleome.org/experiment-sets/4DNES92AU9JR/), and HCT116 WAPL-mAID2 knock-down (HCT116 WAPL KD; auxin-treated HCT116 WAPL-mAID2) (https://data.4dnucleome.org/experiment-sets/4DNES72NE7SL/) by first using the data down sampled in a trio to the lowest fraction read depth as described below, and also normalizing for overall read depth for the sample group of wild type HCT116, HCT116 Rad21 KD, and HCT116 WAPL KD. Counts in each fraction ($S_{i,j}$) were then normalized by sequencing depth by virtue of read per million (RPM) such that $S_{norm,j,50kb\_bin} = S_j, 50_{kb\_bin} / S_{i,j} * 1e6$. Repli-seq arrays were subsequently constructed from RPM bedgraphs to form 16 rows with each row representing an S phase fraction and each column representing a 50 kb bin. The array was smoothed by applying a Gaussian filter and scaled such that each column sums to 100.

We identified IZs with the clustering algorithm BIRCH (https://dsf.berkeley.edu/cs286/papers/birch-sigmod1996.pdf). We assigned each genomic bin to a BIRCH cluster characterized by a centroid. We sorted cluster centroids by the S phase fraction in which the row-wise maximum of the cluster centroid was located. We defined IZs as consecutive (≥2) bins assigned to the same cluster and flanked by neighboring bins that were assigned to clusters associated with cluster centroids whose maxima were located at later rows (i.e later S phase fractions). We empirically determined cluster number of 315 for both WT and Rad21 KD to account for higher noise in Rad21 KD Repli-Seq dataset due to IZ diffusion. IZs are provided in **Supplementary Table 16** and provided on the 4DN portal for all autosomal chromosomes at: HCT116 Rad21 KD (https://data.4dnucleome.org/files-processed/4DNFIGOMS9G7/); wild type HCT116 (https://data.4dnucleome.org/files-processed/4DNFIYO3H24N/).

> Group 4: 16-fraction Repli-seq in wild type HCT116 versus HCT116 WAPL KD

For IZ calling in **Figure 3** and **Extended Data Figure 10d-e** analyses, we normalized wild type HCT116 (https://data.4dnucleome.org/experiment-sets/4DNESNGZM5FG/), HCT116 Rad21-mAID knock-down (HCT116 Rad21 KD; auxin-treated HCT116 Rad21-mAID) (https://data.4dnucleome.org/experiment-sets/4DNES92AU9JR/), and HCT116 WAPL-mAID2 knock-down (HCT116 WAPL KD; auxin-treated HCT116 WAPL-mAID2) (https://data.4dnucleome.org/experiment-sets/4DNES72NE7SL/) by first using the data down sampled in a trio to the lowest fraction read depth as described below, and also normalizing for overall read depth for the sample group of wild type HCT116, HCT116 Rad21 KD, and HCT116 WAPL KD. Counts in each fraction ($S_{i,j}$) were then normalized by sequencing depth by virtue of read per million (RPM) such that $S_{norm,j,50kb\_bin} = S_j, 50_{kb\_bin} / S_{i,j} * 1e6$. Repli-seq arrays were subsequently constructed from RPM bedgraphs to form 16 rows with each row representing an S phase fraction and each column representing a 50 kb bin. The array was smoothed by applying a Gaussian filter and scaled such that each column sums to 100. We identified IZs with the clustering algorithm BIRCH (https://dsf.berkeley.edu/cs286/papers/birch-sigmod1996.pdf). We assigned each genomic bin to a BIRCH

cluster characterized by a centroid. We sorted cluster centroids by the S phase fraction in which the row-wise maximum of the cluster centroid was located. We defined IZs as consecutive (≥2) bins assigned to the same cluster and flanked by neighboring bins that were assigned to clusters associated with cluster centroids whose maxima were located at later rows (i.e later S phase fractions). We empirically determined cluster number of 150 for both WT and WAPL KD. IZs are provided in **Supplementary Table 17** and provided on the 4DN portal for all autosomal chromosomes at: HCT116 WAPL KD (https://data.4dnucleome.org/files-processed/4DNFIDI1QJVA/); wild type HCT116 (https://data.4dnucleome.org/files-processed/4DNFILNNSFMD/)

>Group 5: Sixteen-fraction Repli-seq visualization in HCT116 wild type, Rad21 KD, and WAPL KD

For data visualization in **Figures 2 and 3** and **Extended Data Figure 9**, we normalized wild type HCT116 (https://data.4dnucleome.org/experiment-sets/4DNESNGZM5FG/), HCT116 Rad21-mAID knock-down (HCT116 Rad21 KD; auxin-treated HCT116 Rad21-mAID) (https://data.4dnucleome.org/experiment-sets/4DNES92AU9JR/), and HCT116 WAPL-mAID2 knock-down (HCT116 WAPL KD; auxin-treated HCT116 WAPL-mAID2) (https://data.4dnucleome.org/experiment-sets/4DNES72NE7SL/) by first using the data down sampled in a trio to the lowest fraction read depth as described below, and also normalizing for overall read depth for the sample group of wild type HCT116, HCT116 Rad21 KD, and HCT116 WAPL KD. Counts in each fraction ($S_{i,j}$) were then normalized by sequencing depth by virtue of read per million (RPM) such that $S_{norm,j,50kb\_bin} = S_j, 50_{kb\_bin} / S_{i,j} * 1e6$. Repli-seq arrays were subsequently constructed from RPM bedgraphs to form 16 rows with each row representing an S phase fraction and each column representing a 50 kb bin. The array was smoothed by applying a Gaussian filter and scaled such that each column sums to 100. Links for the data arrays for the read-depth normalized perturbative trio are:

(1) HCT116 WT – read depth normalized down sampled array for visualization: https://data.4dnucleome.org/files-processed/4DNFI6NGWNOG/
(2) HCT116 Rad21 KD – read depth normalized down sampled array for visualization: https://data.4dnucleome.org/files-processed/4DNFI3ZMWG5T/
(3) HCT116 WAPL KD – read depth normalized down sampled array for visualization: https://data.4dnucleome.org/files-processed/4DNFI7MI88QR/

IZ Randomization Test

We computed IZ distance to the nearest boundary for each of the six boundary classes using IZs in Early, Early-mid, and Late (Mid-late + Late) S phase from autosomal chromosomes in hES cells. To create a null set of IZs, we computationally sampled the genome for the same number of random intervals matched by size and A/B compartment distribution to the real IZs. We used only null and real IZ in autosomal regions with sufficient counts for the statistical test and filtered unmappable telomeric/centromeric regions (**Supplementary Table 5**, tab Early IZs, tab Early-mid IZs, tab Late IZs, **Supplementary Table 19**). We computed the test statistic as $d_{real} = (mean\_distance_{null\_IZs} - mean\_distance_{real\_IZs})$. To ascertain the distribution of the test statistic under the null hypothesis, we created a null distribution by permuting/reshuffling the labels on null and real IZs and recomputing the test statistic 150×10^6 times, effectively computing the test statistic as $d_{null} = (mean\_distance_{null\_IZs} - mean\_distance_{null\_IZs})$. Only null and real IZ within 4.5 Mb distance of nearest boundary were used. We computed a one-tailed empirical p-value as the area under the d_{null} test statistic null distribution to the right of the d_{real} value.

Two-fraction Repli-seq of genome edited human iPS and HAP1 cells

Early/Late two-fraction Repli-seq (E/L Repli-seq) in genome edited iPS cells was carried out according to Marchal et al. 2018[74]. Briefly, cells were labelled with BrdU (Sigma Aldrich, Cat# B5002) for 2 hours, subsequently harvested and FACSed into early and late S phase fractions based on the Propidium Iodide

staining profile. BrdU labeled DNA was immunoprecipitated and used for next generation sequencing library preparation. Analysis was performed on the reads generated by sequencing early and late S phase libraries. The log2 ratio of early divided by late S phase was calculated and data were Loess-smoothed (**Figure 4**).

SNS-seq analysis and alignment to domain boundaries

We reanalyzed SNS-seq data from hES cells previously reported by Besnard et al.[51] in the context of the 3D genome boundary classes. SNS-seq dataset was first aligned to hg38 and log2 fold enrichment of signal was calculated over input. The alignment line plots were generated by taking the column mean of a matrix where each row represents a locus centered on the boundary of the described category and each column represents a genomic bin.

Optical Replication Mapping (ORM) data generation and processing

We synchronized HCT116-RAD21-mAID cells in mitosis and treated with auxin as in 16-fraction Repli-seq, except that mitotic cells were released into fresh media containing 10 µg/mL aphidicolin and returned to the incubator for 16 hours to synchronize at G1/S (**Extended Data Figure 7a**). Cells were washed three times with cold PBS to release from the aphidicolin block. Next, cells were nucleofected (Lonza, kit SE, program EN113) in the presence of 40 µM Aminoallyl-dUTP-ATTO-647N (Jena Bioscience, Cat# NU-803-647N) and returned to the incubator to recover overnight. Rad21 knock-down cells were recovered in media containing 500 µM Auxin (HCT116 Rad21-mAID) to maintain protein degradation while HCT116 Rad21 WT cells were recovered in media containing an equivalent volume of DMSO. Live cells were then frozen down in media plus 10% DMSO, stored at -80°C, and shipped to Northwestern Bionano facility as described[75]. IZs were identified in each sample as previously described[75]. Those with a relative peak height (as measured by the difference in signal density between the peak and the edges of the zone) of greater than 2.5% were retained. The size of each IZ was defined by the smallest window that contains at least 30% of ORM signals within each peak. To map ORM pileup at boundaries, we averaged raw ORM data (HCT116 Rad21 WT and HCT116 Rad21 KD) +/- 750 kb from the center of each HCT116 Rad21 WT mitochondrial normalized IZ (**Supplementary Table 15**) that intersects a given boundary class.

HCT116 WT & HCT116 WAPL Knock-Down Hi-C data generation

We treated asynchronous HCT116 WAPL-mAID2 cells with 1 µM 5-Ph-IAA/auxin for 6 hours to knock-down WAPL. As a control, asynchronous HCT116 WAPL-mAID2 cells were treated with an equivalent amount of DMSO. Cells were harvested and fixed in 1% formaldehyde at room temperature for 10 minutes followed by quenching with 0.2 M glycine for 5 minutes at room temperature. Pellets were washed three times with PBS, snap frozen in liquid nitrogen, and stored at -80C. Hi-C library preparation was performed on ~5 million cross-linked cells using the Arima-Hi-C kit (Arima Genomics, Inc., Cat# A510008) according to the manufacturer's protocols. To create sequencing libraries, the purified proximally-ligated DNA was sheared and 200-600 bp DNA fragments were size selected using SPRI beads. The size-selected fragments were then enriched using Enrichment Beads (provided in Arima-Hi-C kit).

Sequencing libraries were prepared with the NEBNext Ultra II Library Prep Kit (NEB, Cat# E7645S) following the manufacturer's protocol. Briefly, 96 ng of purified, size-selected, proximally-ligated Hi-C DNA samples were A-tailed and ligated with Illumina adaptor. Adaptor-ligated DNA was washed (1) 2x on streptavidin beads in 150 µl of wash buffer at 55°C for 2 minutes and (2) 1x in 100 µL of elution buffer at room temperature using an Arima Hi-C kit. DNA was eluted from streptavidin beads by boiling at 98°C for 10 min in a 15 µL elution buffer. Subsequently, the libraries were amplified using the NEBNext

Ultra II DNA Library Prep Kit for Illumina with 8 PCR cycles according to the manufacturer's protocol. Quality and quantity of resulting libraries were evaluated by the DNA high-sensitivity kit on the Agilent Bioanalyzer 2100 (Agilent, Cat# 5067-4626) and Qubit dsDNA HS (Fisher, Cat# Q32851), respectively. Multiplexed libraries were further quantified using the Library Quantification Kit – Illumina/ABI Prism (Kapa Biosystems, Cat# KK4835), followed by 37-cycle paired-end Illumina sequencing on NextSeq500 (Illumina). Hi-C libraries were sequenced with 37 bp pair-end reads.

HCT116 WT & HCT116 WAPL Knock-Down Hi-C data pre-processing

Fastq files for untreated wild type HCT116 (HCT116 WAPL WT; untreated HCT116 WAPL-mAID2) (https://data.4dnucleome.org/experiment-set-replicates/4DNESNSTBMBY/) and HCT116 WAPL KD (HCT116 WAPL KD; auxin-treated HCT116 WAPL-mAID2) (https://data.4dnucleome.org/experiment-set-replicates/4DNES1JP4KZ1/) were processed using step1 of Hi-C Pro (HiC-Pro 2.7.7) to generate validPairs files. This was followed by PCR duplicate removal across reads and replicates. We assembled a raw contact matrix, R, for each chromosome for merged replicates with each chromosome divided into 20 kb non-overlapping bins. Entries, R_{ij}, represent the number of valid read pairs for interactions between the ith and the jth bin on that chromosome (cis contacts only). We then scaled counts in each chromosomal merged raw matrix, R, for both conditions such that average counts per off-diagonal is the same across conditions. We then performed Knight-Ruiz balancing on merged chromosomal scaled matrices per condition using Juicebox (juicer_tools_0.7.5).

IZ width analysis upon WAPL and Rad21 knock-down

We intersected IZs invariant across (1) HCT116 WT and HCT116 Rad21 KD (Group 3) or (2) HCT116 WT and HCT116 WAPL KD (Group 4) with the six classes of HCT116 boundaries. We then plotted invariant IZ widths and then computed two-tailed p-values using the non-parametric Mann-Whitney U test (scipy.stats.mannwhitneyu version 1.7.1).

Vector construction and genome boundary editing of iPS cells

<u>Empty guide vector construction</u>

The chicken beta-actin promoter in pSpCas9-puro (Addgene, Cat# 62988) was replaced with EFS (EF1α short form) promoter. Briefly, a short fragment that contains KpnI, XhoI and NcoI restriction sites generated from annealing two oligos (Forward: CGGGCCCCCTCGAGCTGCAGATATC; Reverse: CATGGATATCTGCAGCTCGAGGGGGCCCGGTAC) was introduced to pSpCas9-puro by KpnI and NcoI digestion and ligation. The EFS promoter from pWPTGFP (Addgene, Cat# 12255) was then inserted to above modified pSpCas9-puro vector between XhoI and NcoI restriction sites, resulting in EF1a-pSpCas9-puro. A vector named Cl3 was created by introducing a GFP to the vector pX330A-1X4 (Addgene, Cat# 58768) to replace the Cas9 gene, generating a smaller vector that can be used for multiplex guides assembly thereafter. The EFS-GFP fragment isolated from pWPTGFP (Addgene, Cat# 12255) was inserted to Cl3 using XhoI and EcoRI digestion, resulting in CI3-GFP. Meanwhile, a short fragment containing SalI and NotI sites generated from annealing two oligos (Forward: GGCCAGCTAGCGTCGACTGTACATAAGC; Reverse: GGCCGCTTATGTACAGTCGACGCTAGCT) was introduced to EF1a-pSpCas9-puro vector at NotI site. The EFS-GFP fragment plus bGH (bovine growth hormone) poly-(A)-signal isolated from Cl3-GFP was inserted to EF1a-pSpCas9-puro using SalI and NotI digestion, resulting in EF1a-pSpCas9-puro-GFP, the final expression vector.

Construction of guides vector for CTCF motifs or IDS big region deletion

Four sgRNAs that target CTCF two motifs (two sgRNAs for each motif) or four sgRNAs that target the IDS region were cloned into CI3, B1 (Addgene, Cat# 58778), B2 (Addgene, Cat# 58779) and B3 (Addgene, Cat# 58780) vectors individually and were assembled to one multiplex guide plasmid as previously described[64]. We excised the four sgRNAs plus four human U6 promoters from the assembled multiplex guide plasmid and inserted to EF1a-pSpCas9-puro-GFP using PciI and KpnI digestion. For control guide, we cloned a scrambled guide (AACCTACGGGCTACGATACG, Addgene, Cat# 70662) into the EF1a-pSpCas9-puro-GFP using PciI and KpnI digestion.

Transfection

Transfection was carried out with Lonza 4D-Nucleofector™ and P3 primary cell 4D nucleofector kit (Lonza, Cat# V4XP-3024) following the manufacturer's instruction. Briefly, 1-2e6 SA3.5 iPS cells were collected and centrifuged at 120 g for 3 minutes at RT. Cell pellet was carefully resuspended in Lonza P3 solution, and quickly added 8 mg plasmid before electroporation using the code CA137. Transfected cells were cultured in mTeSR Plus media (StemCell, Cat# 05825) for four days and subjected to efficiency analysis via flow cytometry cell sorting.

Single cell colony isolation

Four days post transfection, the iPS cells were suspended in Hank's Balanced Salt Solution (HBSS buffer, ThermoFisher, Cat# 14025092) and filtered through 70 µm cell strainer (Corning, Cat# 431751) before single-cell sorting using BD FACSAria fusion cell sorter (BD Bioscience). Sorted cells were cultured in StemFlex media (ThermoFisher, Cat# A3349401) supplemented with 100 U ml^{-1} penicillin-streptomycin (Thermo Fisher, Cat# 15140163) and 1 x RevitaCell™ (Thermo Fisher, Cat# A2644501). The media was changed every 3 days until single cell clones were sufficiently grown. To screen positive genome editing clones, we extracted genomic DNAs from isolated cell clones using GeneJET Genomic DNA Purification Kit (Thermo Fisher, Cat# K0722) and amplified using GoTaq® Green Master Mix (Promega, Cat# M7122) according to manufacturers' protocols. Positive clones were examined by PCR and confirmed by Sanger sequencing.

Four guides for CTCF two motifs (30 kb) deletion

sgRNA sequence that target to CTCF motif 1:
S1U2: AACAAAATAAAGACACCTGC
S1D2: TCCATCGACTGTAGCAACTA
sgRNA sequence that target to CTCF motif 2:
S2U1: AATTAGGAGATGGTATGCAG
S2D1: AGGTACAAATGTCACCTAGA

Four guides for IDS region deletion

IDS loop-U1: ACTCCGGTGAGGTAGCAAGG
IDS loop-U2: AATATGATCCATGTACTACG
IDS loop-D1: TCCGCAGTGAAGAAGCAACA
IDS loop-D2: ACCATCCGGACCAAACGGGG

Primers for detect CTCF 30 kb deletion and Sanger sequencing

CTCF-S1F2: ATCAGCTTTTGCAGCAATCAG
CTCF-S2R1: AGCACATTTCAGTTCAGATGC

Primers for detect IDS 80 kb deletion and Sanger sequencing
IDS-F2: TGGAACATTACCTCCAGTTACTG
IDS-R2: TTAACAGTCAAGGAAAGCAGCC

Published data re-analyzed in this manuscript

>>Hi-C 2.5 data in H1 human ES cells:
https://data.4dnucleome.org/files-processed/4DNFI82R42AD/

>>16 fraction Repliseq on H1-hESC Tier1 cells:
https://data.4dnucleome.org/experiment-sets/4DNESXRBILXJ/

>>Hi-C in untreated HCT116 Rad21-mAID cells:
https://data.4dnucleome.org/files-processed/4DNFIFLDVASC/

>>Hi-C in auxin-treated for 360 minutes HCT116 Rad21-mAID cells:
https://data.4dnucleome.org/files-processed/4DNFILP99QJS/

>>CTCF H1 human ES Cut&Run:
https://data.4dnucleome.org/experiment-set-replicates/4DNES1RQBHPK/

>>Two-fraction Repli-seq for H1 human ES cells:
https://data.4dnucleome.org/files-processed/4DNFIISI1ZA8/

>>CTCF H1 human ES Cut&Run:
https://data.4dnucleome.org/experiment-set-replicates/4DNES1RQBHPK/

>>Rad21 human H9 ES ChIP-seq:
https://www.ncbi.nlm.nih.gov/geo/query/acc.cgi?acc=GSE105028/

>>H1 human ES RNA-seq
https://www.encodeproject.org/experiments/ENCSR537BCG/

>>Rad21 HCT116 ChIP-seq:
https://www.encodeproject.org/experiments/ENCSR000BSB/

>>CTCF HCT116 ChIP-Seq
https://www.encodeproject.org/experiments/ENCSR000BSE/

>>SNS-seq data:
https://www.ncbi.nlm.nih.gov/geo/query/acc.cgi?acc=GSE37757

>>Hi-C WT HAP1:
https://ftp.ncbi.nlm.nih.gov/geo/series/GSE137nnn/GSE137372/suppl/GSE137372_hap1_wt_hic_20000_iced.matrix.gz

https://ftp.ncbi.nlm.nih.gov/geo/series/GSE137nnn/GSE137372/suppl/GSE137372_hap1_wt_hic_20000_ord.bed.gz

(8) Hi-C HAP1 CLONE 21:
https://ftp.ncbi.nlm.nih.gov/geo/series/GSE137nnn/GSE137372/suppl/GSE137372_hap1_clone21_hic_20000_iced.matrix.gz
https://ftp.ncbi.nlm.nih.gov/geo/series/GSE137nnn/GSE137372/suppl/GSE137372_hap1_wt_hic_20000_ord.bed.gz

(9) Hi-C in untreated HCT116 Rad21-mAID cells:
https://data.4dnucleome.org/files-processed/4DNFIFLDVASC/

(10) Hi-C in H1 human ES 2.5
https://data.4dnucleome.org/files-processed/4DNFI82R42AD/

(11) 16 fraction Repliseq on H1-hESC Tier1 cells
https://data.4dnucleome.org/experiment-sets/4DNESXRBILXJ/

Supplementary Figures

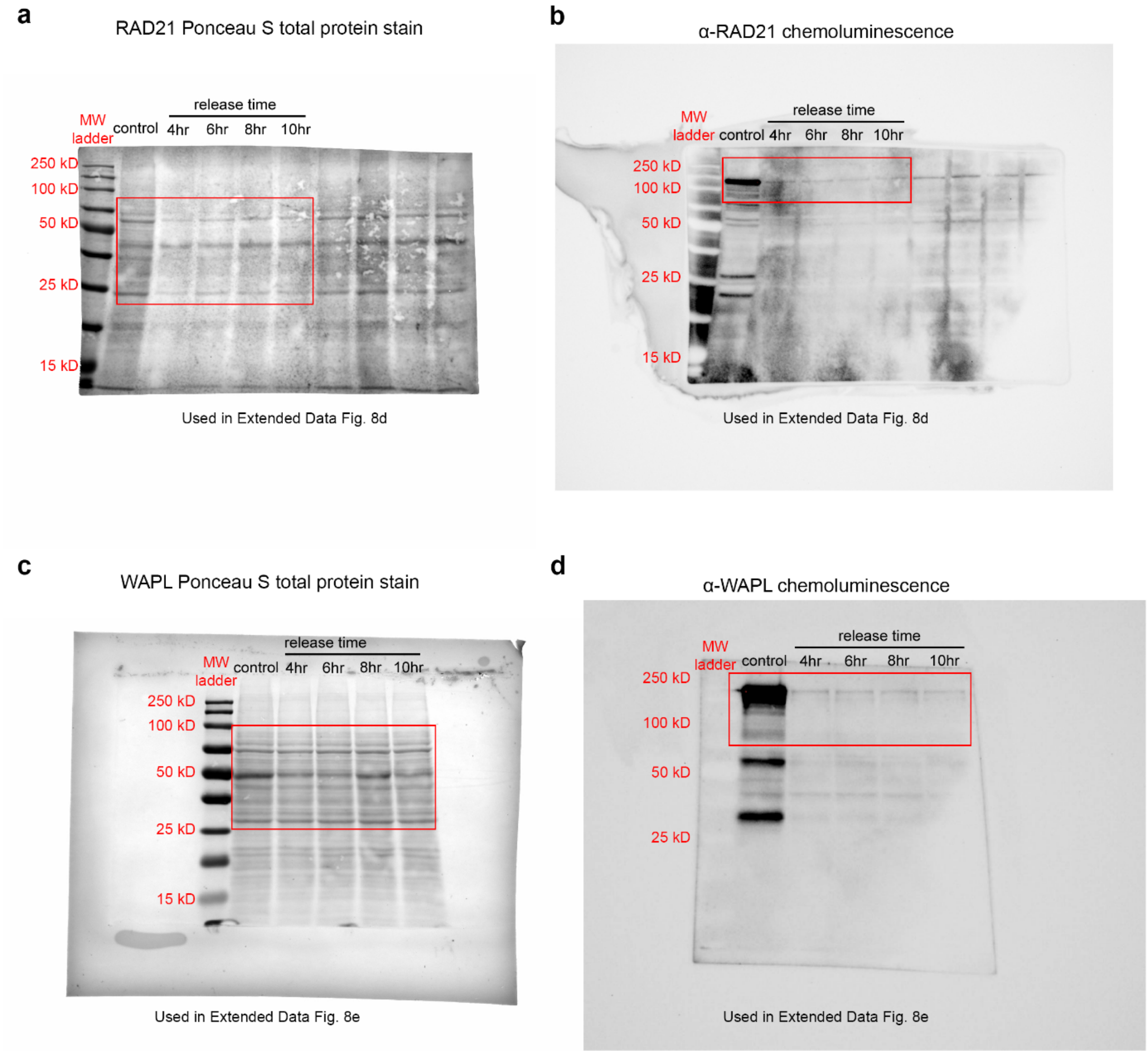

Supplementary Figure 1: Uncropped blots from Extended Data Figure 7. (a) Western blot from Extended data figure 7d for RAD21 protein in HCT116 Rad21-mAID cells for untreated control and timepoints (4, 6, 8, and 10 hours) after auxin treatment post mitotic shake off. **(b)** Ponceau S stain of western blot in **Supplementary Figure 1a** showing total protein. **(c)** Western blot from Extended data figure 7e for WAPL protein in HCT116 WAPL-mAID2 cells for untreated control and timepoints (4, 6, 8, and 10 hours) after auxin treatment post mitotic shake off. **(b)** Ponceau S stain of western blot in **Supplementary Figure 1c** showing total protein.

Supplementary References

51. Besnard, E. et al. Unraveling cell type-specific and reprogrammable human replication origin signatures associated with G-quadruplex consensus motifs. *Nat Struct Mol Biol* **19**, 837-844 (2012).
52. Akgol Oksuz, B. et al. Systematic evaluation of chromosome conformation capture assays. *Nat Methods* **18**, 1046-1055 (2021).
53. Norton, H.K. et al. Detecting hierarchical genome folding with network modularity. *Nat Methods* **15**, 119-122 (2018).
54. Sun, J.H. et al. Disease-Associated Short Tandem Repeats Co-localize with Chromatin Domain Boundaries. *Cell* (2018).
55. Zhang, H. et al. Chromatin structure dynamics during the mitosis-to-G1 phase transition. *Nature* **576**, 158-162 (2019).
56. Sun, J.H. et al. Disease-Associated Short Tandem Repeats Co-localize with Chromatin Domain Boundaries. *Cell* **175**, 224-238 e215 (2018).
57. Beagan, J.A. & Phillips-Cremins, J.E. On the existence and functionality of topologically associating domains. *Nat Genet* **52**, 8-16 (2020).
58. Rao, S.S.P. et al. Cohesin Loss Eliminates All Loop Domains. *Cell* **171**, 305-320 e324 (2017).
59. Phillips-Cremins, J.E. Unraveling architecture of the pluripotent genome. *Curr Opin Cell Biol* **28**, 96-104 (2014).
60. Norton, H.K. & Phillips-Cremins, J.E. Crossed wires: 3D genome misfolding in human disease. *J Cell Biol* **216**, 3441-3452 (2017).
61. Rao, S.S. et al. A 3D map of the human genome at kilobase resolution reveals principles of chromatin looping. *Cell* **159**, 1665-1680 (2014).
62. Fernandez, L.R., Gilgenast, T.G. & Phillips-Cremins, J.E. 3DeFDR: statistical methods for identifying cell type-specific looping interactions in 5C and Hi-C data. *Genome Biol* **21**, 219 (2020).
63. Beagan, J.A. et al. Three-dimensional genome restructuring across timescales of activity-induced neuronal gene expression. *Nat Neurosci* **23**, 707-717 (2020).
64. Kim, J.H. et al. LADL: light-activated dynamic looping for endogenous gene expression control. *Nat Methods* **16**, 633-639 (2019).
65. Kim, J.H. et al. 5C-ID: Increased resolution Chromosome-Conformation-Capture-Carbon-Copy with in situ 3C and double alternating primer design. *Methods* **142**, 39-46 (2018).
66. Zhang, D. et al. Alteration of genome folding via contact domain boundary insertion. *Nat Genet* **52**, 1076-1087 (2020).
67. Gilgenast, T.G. & Phillips-Cremins, J.E. Systematic Evaluation of Statistical Methods for Identifying Looping Interactions in 5C Data. *Cell Syst* **8**, 197-211 e113 (2019).
68. Beagan, J.A. et al. YY1 and CTCF orchestrate a 3D chromatin looping switch during early neural lineage commitment. *Genome Res* **27**, 1139-1152 (2017).
69. Crane, E. et al. Condensin-driven remodelling of X chromosome topology during dosage compensation. *Nature* **523**, 240-244 (2015).
70. Klein, K.N. et al. Replication timing maintains the global epigenetic state in human cells. *Science* **372**, 371-378 (2021).
71. Saito, Y. & Kanemaki, M.T. Targeted Protein Depletion Using the Auxin-Inducible Degron 2 (AID2) System. *Curr Protoc* **1**, e219 (2021).
72. Oldach, P. & Nieduszynski, C.A. Cohesin-Mediated Genome Architecture Does Not Define DNA Replication Timing Domains. *Genes (Basel)* **10** (2019).
73. Zhao, P.A., Sasaki, T. & Gilbert, D.M. High-resolution Repli-Seq defines the temporal choreography of initiation, elongation and termination of replication in mammalian cells. *Genome Biol* **21**, 76 (2020).

74. Marchal, C. et al. Genome-wide analysis of replication timing by next-generation sequencing with E/L Repli-seq. *Nat Protoc* **13**, 819-839 (2018).
75. Wang, W. et al. Genome-wide mapping of human DNA replication by optical replication mapping supports a stochastic model of eukaryotic replication. *Mol Cell* **81**, 2975-2988 e2976 (2021).
