# Supplementary material for: Cohesin-mediated loop anchors confine the locations of human replication origins
Source: Nature. 2022 Jun 8;606(7915):812–9. doi: 10.1038/s41586-022-04803-0 (PMC9217744; doi:10.1038/s41586-022-04803-0)
Supplement: Supplementary file 2 — Reporting Summary [file 41586_2022_4803_MOESM2_ESM.pdf]

Corresponding author(s): *Double-blind peer review submissions: write DBPR and your manuscript number here instead of author names.*

Last updated by author(s): 2/4/2022

Reporting Summary

Nature Research wishes to improve the reproducibility of the work that we publish. This form provides structure for consistency and transparency in reporting. For further information on Nature Research policies, see our Editorial Policies and the Editorial Policy Checklist.

Statistics

For all statistical analyses, confirm that the following items are present in the figure legend, table legend, main text, or Methods section.

n/a	Confirmed	
☐	☒	The exact sample size (*n*) for each experimental group/condition, given as a discrete number and unit of measurement
☐	☒	A statement on whether measurements were taken from distinct samples or whether the same sample was measured repeatedly
☐	☒	The statistical test(s) used AND whether they are one- or two-sided<br>*Only common tests should be described solely by name; describe more complex techniques in the Methods section.*
☒	☐	A description of all covariates tested
☐	☒	A description of any assumptions or corrections, such as tests of normality and adjustment for multiple comparisons
☒	☐	A full description of the statistical parameters including central tendency (e.g. means) or other basic estimates (e.g. regression coefficient) AND variation (e.g. standard deviation) or associated estimates of uncertainty (e.g. confidence intervals)
☐	☒	For null hypothesis testing, the test statistic (e.g. F, t, r) with confidence intervals, effect sizes, degrees of freedom and P value noted<br>*Give P values as exact values whenever suitable.*
☒	☐	For Bayesian analysis, information on the choice of priors and Markov chain Monte Carlo settings
☒	☐	For hierarchical and complex designs, identification of the appropriate level for tests and full reporting of outcomes
☒	☐	Estimates of effect sizes (e.g. Cohen's *d*, Pearson's *r*), indicating how they were calculated

Our web collection on statistics for biologists contains articles on many of the points above.

Software and code

Policy information about availability of computer code

Data collection	Juicebox (juicer_tools_0.7.5.jar) was used for extracting hic chromosome matrices from .hic files with conversion to .npz sparse matrices. Cooler package in python (cooler 0.8.10) was used for extraction of hic chromosome matrices from .cool files with conversion to .npz sparse matrices
Data analysis	Domain calls were made with 3dnetmod from https://bitbucket.org/creminslab/cremins_lab_tadsubtad_calling_pipeline_11_6_2021/. loops on hic were called from custom python code based on HICCUPs approach from Aiden and colleagues available https://bitbucket.org/creminslab/cremins_lab_loop_calling_pipeline_11_6_2021/. bowtie version 0.12.7 and samtools version 1.2 were used for Chipseq and Cut&Run fastq processing. MACS2 2.1.1.20160309 was used for peak calls. bedtools 2.15.0 was used for intersection of peaks with domain boundaries. opencv-python 4.2.0.32 (cv2 4.2.0) was used for APA pileup of domains. linalg class of numpy (numpy 1.16.6) was used for eigenvector decomposition and compartment calling in hic matrices. Custom scripts in python were used to access domain layer hierarchy and domain intersection with compartment and dots and subsequent boundary classification as well as IZ to closest boundary permutation test. For repliseq, the BIRCH clustering algorithm was used for IZ determination.

For manuscripts utilizing custom algorithms or software that are central to the research but not yet described in published literature, software must be made available to editors and reviewers. We strongly encourage code deposition in a community repository (e.g. GitHub). See the Nature Research guidelines for submitting code & software for further information.

Data

Policy information about availability of data

All manuscripts must include a data availability statement. This statement should provide the following information, where applicable:

- Accession codes, unique identifiers, or web links for publicly available datasets
- A list of figures that have associated raw data
- A description of any restrictions on data availability

A full Data Access inventory is listed on p. 12 of the manuscript:

Data Availability Statement

>>All new raw data created in this manuscript has been uploaded to the 4D Nucleome portal and will be freely released for full distribution to the scientific community.

(1) Group 1: Sixteen-fraction Repli-seq data for H1 human ES cells:
- H1 human ES raw fastq: https://data.4dnucleome.org/experiment-sets/4DNESXRBILXJ/
- H1 human ES read depth normalized array for visualization: https://data.4dnucleome.org/files-processed/4DNFIEEYFQ7C/
- H1 human ES read depth scaled normalized array for IZ calls: https://data.4dnucleome.org/files-processed/4DNFI3N8GHKR/
- H1 human ES Early, Early-mid, Late IZs on read depth normalized array: https://data.4dnucleome.org/files-processed/4DNFIRF7WZ3H/

(2) Group 2: Sixteen-fraction Repli-seq data for Wild Type HCT116 cells:
- WT HCT116 raw fastq: https://data.4dnucleome.org/experiment-sets/4DNESNGZM5FG/
- WT HCT116 mitochondria normalized array for IZ calls: https://data.4dnucleome.org/files-processed/4DNFIPIQTMJ9/
- WT HCT116 Early, Early-mid, Late IZs on mitochondria normalized array: https://data.4dnucleome.org/files-processed/4DNFI95K53YS/

(3) Group 3: Sixteen-fraction Repli-seq data for wild type and cohesin knock-down HCT116 pairing:
- Rad21 knock-down HCT116 raw: https://data.4dnucleome.org/experiment-sets/4DNES92AU9JR/
- Rad21 knock-down HCT116 read depth normalized down sampled array for IZ calls: https://data.4dnucleome.org/files-processed/4DNFI3ZMWG5T/
- Rad21 knock-down HCT116 Early, Early-mid, Late IZs called on the read depth normalized down sampled array: https://data.4dnucleome.org/files-processed/4DNFIGOMS9G7/
- WT HCT116 raw fastq: https://data.4dnucleome.org/experiment-sets/4DNESNGZM5FG/
- WT HCT116 read depth normalized down sampled array for IZ calls: https://data.4dnucleome.org/files-processed/4DNFI6NGWNOG/
- WT HCT116 Early, Early-mid, Late IZs called on the read depth normalized down sampled array: https://data.4dnucleome.org/files-processed/4DNFIYO3H24N/

(4) Group 4: Sixteen-fraction Repli-seq data for wild type and WAPL knock-down HCT116 pairing:
- WAPL knock-down HCT116 raw: https://data.4dnucleome.org/experiment-sets/4DNES72NE7SL/
- WAPL knock-down HCT116 read depth normalized down sampled array for IZ calls: https://data.4dnucleome.org/files-processed/4DNFI7MI88QR/
- WAPL knock-down HCT116 Early, Early-mid, Late IZs called on the read depth normalized down sampled array: https://data.4dnucleome.org/files-processed/4DNFIDI1QJVA/
- WT HCT116 raw fastq: https://data.4dnucleome.org/experiment-sets/4DNESNGZM5FG/
- WT HCT116 read depth normalized down sampled array for IZ calls: https://data.4dnucleome.org/files-processed/4DNFI6NGWNOG/
- WT HCT116 Early, Early-mid, Late IZs called on the read depth normalized down sampled array: https://data.4dnucleome.org/files-processed/4DNFILNNSFMD/

(5) Group 5: Sixteen-fraction Repli-seq data visualization
- WT HCT116 read depth normalized down sampled array for visualization: https://data.4dnucleome.org/files-processed/4DNFI6NGWNOG/
- Rad21 knock-down HCT116 read depth normalized down sampled array for visualization: https://data.4dnucleome.org/files-processed/4DNFI3ZMWG5T/
- WAPL knock-down HCT116 read depth normalized down sampled array for visualization: https://data.4dnucleome.org/files-processed/4DNFI7MI88QR/

(6) Hi-C for wild type and WAPL knock-down HCT116 pairing:
- WAPL KD HCT116 raw Hi-C: https://data.4dnucleome.org/experiment-set-replicates/4DNES1JP4KZ1/
- WAPL KD HCT116 normalized balanced Hi-C matrices: https://data.4dnucleome.org/files-processed/4DNFIY5939F3/
- WAPL KD HCT116 Loops: https://data.4dnucleome.org/files-processed/4DNFILP7BD5H/
- WT HCT116 raw Hi-C: https://data.4dnucleome.org/experiment-set-replicates/4DNESNSTBMBY/
- WT HCT116 normalized balanced Hi-C matrices: https://data.4dnucleome.org/files-processed/4DNFI5MR78O6/
- WT HCT116 Loops: https://data.4dnucleome.org/files-processed/4DNFIOQLL854/

(7) Two-fraction Repli-seq data for human iPS wild type and two CRISPR engineered lines: raw data and processed log2(Early/Late) from three conditions
- WT human iPS line Raw Data: https://data.4dnucleome.org/experiment-sets/4DNESDYES9QD/
- WT human iPS line log2(Early/Late): https://data.4dnucleome.org/files-processed/4DNFI5WEY784/
- human engineered clone 1 80kb IZ deletion iPS line Raw Data: https://data.4dnucleome.org/experiment-sets/4DNESE3WCUAQ/
- human engineered clone 1 80kb IZ deletion iPS line log2(Early/Late): https://data.4dnucleome.org/files-processed/4DNFIZMB415V/
- human engineered clone 2 30kb control deletion iPS line Raw Data: https://data.4dnucleome.org/experiment-sets/4DNES66YWJU7/
- human engineered clone 2 30kb control deletion iPS line log2(Early/Late): https://data.4dnucleome.org/files-processed/4DNFIWDMF7HW/

(8) 5C data for human IPS wild type and two engineered lines:
primer bed file, raw heatmaps and processed heatmaps from three conditions
- WT human iPS line Raw Data: https://data.4dnucleome.org/experiment-set-replicates/4DNESLRDUPZ6/
- WT human iPS line balanced 5C data: replicate 1: https://data.4dnucleome.org/files-processed/4DNFIXM8V3ZB/; replicate 2: https://data.4dnucleome.org/files-processed/4DNFIDB6M1ZN/
- WT human engineered clone 1 80kb boundary deletion iPS line Raw Data: https://data.4dnucleome.org/experiment-set-replicates/4DNES39F1QWU/
- WT human engineered clone 1 80kb boundary deletion iPS line balanced 5C data: https://data.4dnucleome.org/files-processed/4DNFIA8P94BX/
- WT human engineered clone 2 30kb control deletion iPS line Raw Data: https://data.4dnucleome.org/experiment-set-replicates/4DNES3PDMUHG/
- WT human engineered clone 2 30kb control deletion iPS line balanced 5C data: replicate 1: https://data.4dnucleome.org/files-processed/4DNFI7WZYRHP/;

replicate 2: https://data.4dnucleome.org/files-processed/4DNFI7V4VXAQ/

>> 4D Nucleome data analyzed for this manuscript:
(1) Hi-C 2.5 data in H1 human ES cells:
https://data.4dnucleome.org/files-processed/4DNFI82R42AD/

(2) 16 fraction Repliseq on H1-hESC Tier1 cells:
https://data.4dnucleome.org/experiment-sets/4DNESXRBILXJ/

(3) Hi-C in untreated HCT116 Rad21-mAID cells:
https://data.4dnucleome.org/files-processed/4DNFIFLDVASC/

(4) Hi-C in auxin-treated for 360 minutes HCT116 Rad21-mAID cells:
https://data.4dnucleome.org/files-processed/4DNFILP99QJS/

(5) CTCF H1 human ES Cut&Run:
https://data.4dnucleome.org/experiment-set-replicates/4DNES1RQBHPK/

(6) Two-fraction Repli-seq for H1 human ES cells:
https://data.4dnucleome.org/files-processed/4DNFIISI1ZA8/

>>Processed data files for all Figures are provided as Supplementary Tables.

>>Reanalyzed data:
(1) CTCF H1 human ES Cut&Run:
https://data.4dnucleome.org/experiment-set-replicates/4DNES1RQBHPK/

(2) Rad21 human H9 ES ChIP-seq:
https://www.ncbi.nlm.nih.gov/geo/query/acc.cgi?acc=GSE105028/

(3) H1 human ES RNA-seq
https://www.encodeproject.org/experiments/ENCSR537BCG/

(4) Rad21 HCT116 ChIP-seq:
https://www.encodeproject.org/experiments/ENCSR000BSB/

(5) CTCF HCT116 ChIP-Seq
https://www.encodeproject.org/experiments/ENCSR000BSE/

(6) SNS-seq data:
https://www.ncbi.nlm.nih.gov/geo/query/acc.cgi?acc=GSE37757

(7) Hi-C WT HAP1:
https://ftp.ncbi.nlm.nih.gov/geo/series/GSE137nnn/GSE137372/suppl/GSE137372_hap1_wt_hic_20000_iced.matrix.gz
https://ftp.ncbi.nlm.nih.gov/geo/series/GSE137nnn/GSE137372/suppl/GSE137372_hap1_wt_hic_20000_ord.bed.gz

(8) Hi-C HAP1 CLONE 21:
https://ftp.ncbi.nlm.nih.gov/geo/series/GSE137nnn/GSE137372/suppl/GSE137372_hap1_clone21_hic_20000_iced.matrix.gz
https://ftp.ncbi.nlm.nih.gov/geo/series/GSE137nnn/GSE137372/suppl/GSE137372_hap1_wt_hic_20000_ord.bed.gz

(9) Hi-C in untreated HCT116 Rad21-mAID cells:
https://data.4dnucleome.org/files-processed/4DNFIFLDVASC/

(10) Hi-C in H1 human ES 2.5
https://data.4dnucleome.org/files-processed/4DNFI82R42AD/

(11) 16 fraction Repliseq on H1-hESC Tier1 cells
https://data.4dnucleome.org/experiment-sets/4DNESXRBILXJ/

>>Data links
(1) ORM Data
NCBI BioProject database accession PRJNA788726 http://genome.ucsc.edu/s/dsaulebe/ORM%20data%20HCT116

(2) Two-fraction Repli-seq data for Blobel engineered lines: raw data and processed log2(Early/Late) from three conditions
https://www.ncbi.nlm.nih.gov/geo/query/acc.cgi?acc=GSE190117

Field-specific reporting

Please select the one below that is the best fit for your research. If you are not sure, read the appropriate sections before making your selection.

☒ Life sciences ☐ Behavioural & social sciences ☐ Ecological, evolutionary & environmental sciences

For a reference copy of the document with all sections, see nature.com/documents/nr-reporting-summary-flat.pdf

Life sciences study design

All studies must disclose on these points even when the disclosure is negative.

Sample size	N/A
Data exclusions	N/A
Replication	The early IZ enrichment findings were reproduced with both 16 fraction Repli-seq and SNS-seq. The early IZ peturbation result upon cohesin knock-down was reproduced with two-fraction Repli-seq, sixteen-fraction Repli-seq, and single-fraction early S phase BrdU labeling and pull-down.
Randomization	N/A
Blinding	N/A

Reporting for specific materials, systems and methods

We require information from authors about some types of materials, experimental systems and methods used in many studies. Here, indicate whether each material, system or method listed is relevant to your study. If you are not sure if a list item applies to your research, read the appropriate section before selecting a response.

Materials & experimental systems

n/a	Involved in the study	
☒	☐	Antibodies
☐	☒	Eukaryotic cell lines
☒	☐	Palaeontology and archaeology
☒	☐	Animals and other organisms
☒	☐	Human research participants
☒	☐	Clinical data
☒	☐	Dual use research of concern

Methods

n/a	Involved in the study	
☒	☐	ChIP-seq
☒	☐	Flow cytometry
☒	☐	MRI-based neuroimaging

Eukaryotic cell lines

Policy information about cell lines

Cell line source(s)	H1 human ES cells, HCT116 cell line
Authentication	All cell lines and their stocks and their culture protocols have been authenticated by the 4D Nucleome and 4D Nucleome standards were used.
Mycoplasma contamination	The lines have been verified via the 4D Nucleome.
Commonly misidentified lines (See ICLAC register)	N/A
